# A MicroRNA Signature Predicts Survival in Early Stage Small-Cell Lung Cancer Treated with Surgery and Adjuvant Chemotherapy

**DOI:** 10.1371/journal.pone.0091388

**Published:** 2014-03-17

**Authors:** Nan Bi, Jianzhong Cao, Yongmei Song, Jie Shen, Wenyang Liu, Jing Fan, Jie He, Yuankai Shi, Xun Zhang, Ning Lu, Qimin Zhan, Luhua Wang

**Affiliations:** 1 Department of Radiation Oncology, Cancer Hospital and Institute, Chinese Academy of Medical Sciences and Peking Union Medical College, Beijing, China; 2 State Key Laboratory of Molecular Oncology, Cancer Hospital and Institute, Chinese Academy of Medical Sciences and Peking Union Medical College, Beijing, China; 3 Department of Thoracic Surgery, Cancer Hospital and Institute, Chinese Academy of Medical Sciences and Peking Union Medical College, Beijing, China; 4 Department of Medical Oncology, Cancer Hospital and Institute, Chinese Academy of Medical Sciences and Peking Union Medical College, Beijing, China; 5 Department of Pathology, Cancer Hospital and Institute, Chinese Academy of Medical Sciences and Peking Union Medical College, Beijing, China; IPMC, CNRS UMR 7275 UNS, France

## Abstract

Small-cell lung cancer (SCLC) is one of the most aggressive cancers, yet the molecular mechanisms underlying its devastating clinical outcome remain elusive. In this study, we investigated whether microRNA (miRNA) expression profiles can predict the clinical outcomes of SCLC patients. A total of 82 patients with limited SCLC, who were treated with surgical resection and adjuvant chemotherapy, were enrolled in this study. First, we surveyed the expression of 924 miRNAs from 42 SCLC patients to discover survival-relevant miRNAs and develop prognostic models, which were then validated in an independent cohort of 40 cases using quantitative real-time PCR. We found that the miR-150/miR-886-3p signature was significantly correlated with the overall survival (OS) of SCLC patients (p = 0.02) in the training set, and both miRNA expression levels were much lower in the SCLC samples than normal lung samples. The miRNA signature also proved to be a significant predictor of survival in the validation set. Patients with high-risk miRNA signatures had poor overall survival (p = 0.005) and progression-free survival (p = 0.017) compared with those with low-risk scores. These findings retained statistical significance after adjusting for age, gender and smoking status (HR: 0.26, 95%: CI 0.10–0.69, p = 0.007), which suggested it may be an independent predictor of survival. In summary, we developed a prognostic miR-150/miR-886-3p signature and validated expression in an independent dataset of resectable SCLC. These preliminary results indicated that miRNAs may serve as promising molecular prognostic markers and new therapeutic targets for SCLC.

## Introduction

Lung cancer remains the leading cause of death in the world, and small-cell lung cancer (SCLC) accounts for 15–25% of cases [1]. Clinically, SCLC is distinguished from non-small cell lung cancer (NSCLC) by rapid tumor growth and the early onset of metastases. Unlike in most Western countries, SCLC rates are still increasing in China, where smoking prevalence also continues to increase. Although SCLC is considered highly responsive to chemotherapy and radiotherapy, relapse often occurs within two years and the overall survival (OS) beyond five years is approximately 3%–8%. To improve clinical outcomes, new drugs have been developed, but it is discouraging that the survival for both localized and advanced SCLC has plateaued in the past 20 years.

SCLC is thought to carry a variety of molecular abnormalities that are quite different from NSCLC. For example, TP53 mutations, Rb inactivation and c-Myc amplification are common in SCLC compared to NSCLC [2], [3]. In contrast, epidermal growth factor receptor (EGFR) mutations, K-ras mutations and P16 inactivation are frequently found in NSCLC, but not in SCLC [2]–[4]. However, the underlying mechanisms by which SCLC rapidly progresses remain to be defined, and the identification of these mechanisms could promote the development of novel therapeutic agents and improve the management of this challenging disease.

MicroRNAs (miRNAs) are a class of noncoding RNAs that are approximately 22 nucleotides long and are post-transcriptional regulators of gene expression. Because of their fundamental role in development and differentiation, involvement in the biological mechanisms underlying tumorigenesis and progression, as well as low complexity, stability, and easy detection, miRNAs represent a promising class of tissue- and blood-based biomarkers of cancer.

A number of researchers have demonstrated that aberrant miRNA expression is closely related to the development and progression of many cancers, behaving as tumor suppressors or oncogenes [5]–[10]. Recent data from multiple studies strongly support the potential of microRNAs as biomarkers for NSCLC [11]–[17]. In addition, altered microRNA expression is also associated with tumor progression and survival in NSCLC cancer patients. A global miRNA expression pattern assessed using microarray based analysis has been established, and several NSCLC “miRNA signatures” have been proposed for improved molecular staging and classification of NSCLC [18]–[21]. However, the clinical significance of miRNAs in SCLC has not been well established.

To investigate the role of aberrant miRNA expression profiles in SCLC, we first surveyed 924 known miRNAs in formalin-fixed paraffin-embedded (FFPE) specimens from 42 patients and identified a dual-miRNA signature to predict the clinical outcome of early stage SCLC patients treated with surgical resection followed by adjuvant chemotherapy. This association was further validated with quantitative reverse transcriptase polymerase chain reaction (qRT-PCR) analysis of 40 additional patient specimens, and the prognostic value of the signature appears to be independent of age, gender and smoking status in multivariate Cox regression analysis.

## Materials and Methods

### Ethics Statement

This retrospective study was approved by the Institutional Review Board of the Cancer Hospital and Institute of Chinese Academy of Medical Sciences and Peking Union Medical College. All of the patient records used in this study were made anonymous and de-identified before analysis.

### Patients and samples

We retrospectively studied formalin-fixed paraffin-embedded (FFPE) specimens from patients who were diagnosed with resectable limited SCLC and underwent surgery followed by adjuvant chemotherapy (platinum-based regimens such as cisplatin/etoposide and carboplatin/etoposide) at the Cancer Hospital and Institute of Chinese Academy of Medical Sciences (Beijing, China) between January 2000 and December 2005. All of the patients had an ECOG performance status of 0–1, and patients with mixed small-cell/large-cell carcinoma or combined small cell carcinoma were excluded. In total, 82 SCLC patient samples were analyzed, among which 42 samples were used as a screening set to identify miRNAs associated with survival using a miRNA microarray. The selected miRNAs were then validated in the remaining 40 patients using real-time PCR. Hematoxylin and eosin (H&E) sections of all of the samples were reviewed by a pathologist to confirm the diagnosis. Three normal lung tissue (NL) samples were obtained from macroscopically uninvolved areas 2–3 cm away from benign nodules of patients who underwent surgical resection. All of the NL tissues were histopathologically assessed and were morphologically normal.

### RNA extraction

Low-molecular-weight RNA was isolated from total RNA, which was extracted with TRIZOL, using a PEG solution precipitation method [22]. In brief, to obtain low molecular weight RNA, total RNA was precipitated with equal amounts of PEG solution, and the supernatant was precipitated using isopropanol. All of the RNA extracts were assessed and showed no signs of RNA degradation.

### miRNA microarray analysis

Microarray analysis was performed as previously described [23]. Briefly, RNA was labeled using the T4 RNA ligase labeling method. Hybridization was performed using a custom microRNA microarray panel (CapitalBio, Beijing, China), which included probes in triplicate for 924 mature human and mouse miRNA sequences and eight short oligonucleotides that possessed no homology to any known RNA sequence as external controls. The hybridized arrays were scanned with a LuxScan 10K-A laser confocal scanner, and the images obtained were then analyzed using the LuxScan 3.0 software (CapitalBio, Beijing, China) [6]. For data processing, only data for the human miRNAs (546 of 924) was examined. First, the raw data were pre-processed to filter out miRNAs with maximum intensities less than 300 to reduce bias in the normalization step. After filtration, 456 (83.5%) of 546 miRNAs were included for further analysis. Then, the average values of the replicate spots for each miRNA were background-subtracted, and the signals were normalized using the median center tool for genes with Cluster 3.0 software.

Hazard ratios (HR) from univariate Cox regression analysis, a standard method in biostatistics for examining survival data that minimizes bias by simply removing censored patients, were used to identify which miRNAs were correlated with the OS of the patients [21]. We used a P-value of 0.1 as the cutoff for gene selection. A risk score for the miRNA signature survival prognosis was calculated according to a combination of the expression level of the miRNA weighted by the regression coefficient derived by univariate Cox regression analysis [21], [24], [25]. A class comparison with the nonparametric Mann-Whitney U test was also performed to identify differently expressed miRNAs between the 42 SCLC samples and the three NL samples.

All of the raw data were deposited in a MIAME compliant database (ArrayExpress, GEO: GSM678225 - GSM678266).

### miRNA quantitative real-time RT-PCR

cDNA was prepared from 100 ng of total RNA with specific primers for miR-886-3p, miR-150 or U6 as the internal control. For the detection of mature miRNAs, quantitative miRNA RT-PCR was performed using the stem-loop qRT-PCR method [26]. The oligos used included the miRNA common reverse primer, GTGCAGGGTCCGAGGT; miR-150, TCTCCCAACCCTTGTACCAGTG; miR-150 RT primer, GTCGTATCCAGTGCAGGGTCCGAGGTATTCGCACTGGATACGACCACTGG; miR-150 forward primer, GTCTCCCAACCCTTGTACCA; miR-886-3p, CGCGGGTGCTTACTGACCCTT; miR-886-3p RT primer, GTCGTATCCAGTGCAGGGTCCGAGGTATTCGCACTGGATACGACAAGGGT; miR-886-3p forward primer, CACGCGGGTGCTTACTGAC; U6 forward primer, CTCGCTTCGGCAGCACA; and U6 reverse primer, AACGCTTCACGAATTTGCGT. qRT-PCR was performed using an ABI Prizm 7300 Sequence Detection System with LightCycler DNA Master SYBR Green I mix (Roche Molecular Biochemicals, Mannheim, Germany) following the manufacturer's instructions. The level of miRNAs was determined using the 2^–ΔΔCt^ method with SDS 1.3 software, normalized to the level of the internal control, U6, and plotted for inter-cellular comparison.

### Endpoints

The primary endpoint of this study was OS. Secondary endpoints included progression-free survival (PFS), local-regional control (LRC), and distant metastasis-free survival (DMFS). OS was calculated from the date of diagnosis to death from any cause or the last known date that the patient was alive. PFS was defined as the duration from the date of diagnosis to the date of failure either in the thorax or at distant sites, the date of death from any cause, or the date of the last patient follow-up.

LRC was calculated from the date of diagnosis to the first local-regional (thorax) recurrence, and DMFS was calculated from the date of diagnosis to the first distant metastasis, the date of death from any cause, or the date of the last patient follow-up.

### Statistical analysis

The clinical characteristics of different groups were compared using the independent student's t test, χ^2^ test, or Fisher's exact test. The correlation between the microarray data and the qRT-PCR data was analyzed with the Spearman correlation. OS, PFS, LRC and DMFS were estimated with the Kaplan-Meier method and log-rank test. HRs were calculated with Cox regression analysis. A multivariate Cox regression model was used to test if the miRNA signature was an independent prognostic factor when adjusted for age, sex, and smoking status. All of the analyses were performed using the SPSS software package (version 11.5, SPSS Inc., Chicago, IL). A two-sided p-value of less than 0.05 was considered to be statistically significant.

## Results

The clinical characteristics of the 82 SCLC patients are listed in Table 1. The median follow-up time was 57.2 months. Forty-four patients are still alive. Forty-two patients had recurrent disease, and the median time to a diagnosis of relapse was 12.3 months.

**Table 1:** Clinical characteristics of the LD-SCLC patients.

Characteristic	Training set	Testing set	Total
	(N = 42)	(N = 40)	(n = 82)
**Age, years**			
Median	60.1	55.0	57
Range	33.0–77.0	33.0–73.0	33.0–77.0
**Sex**			
Male (%)	30 (71.4)	32 (80.0)	62 (75.6)
Female (%)	12 (28.6)	8 (20.0)	20 (24.4)
**Smoking status**			
Current/former smokers (%)	30 (71.4)	29 (72.5)	59 (72.0)
Never smokers (%)	12 (28.6)	11 (27.5)	23 (28.0)
**No. of ChT cycle (Platinum-based)**			
2–4	24	19	33
4–6	17	13	30
>6	1	8	9
**Median OS, months**	56.0	67.4	67.4
**Median PFS, months**	NR	44.6	47.7
**Median LRC, months**	NR	NR	NR
**Median DMFS, months**	NR	60.0	60.0

LD, limited stage disease; SCLC, small cell lung cancer; No, number; ChT, chemotherapy; OS, overall survival; PFS, progression-free survival; LRC, local-regional control; DMFS, distant metastasis-free survival; NR, not reached.

**Figure 1:**
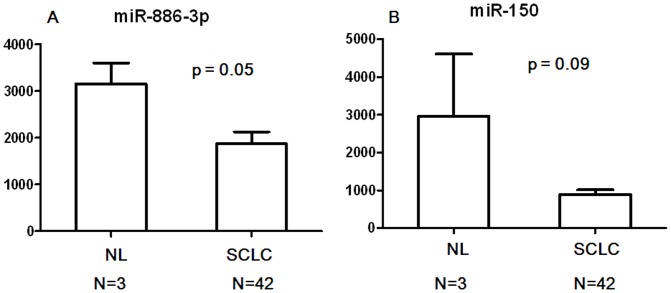
Comparison of expression levels of miR-886-3p and miR-150 between SCLC tumors and normal lung tissues (NL).

In the training set, we identified two miRNAs, miR-150 and miR-886-3p, that were associated with poor OS using the Cox proportional hazard regression model. Both of them were protective (Table 2). The comparison between NL and SCLC tissues also verified that miR-150 and miR-886-3p expression levels in SCLC tumors were much lower than in NL samples (Fig 1).

**Table 2:** microRNAs associated with overall survival in SCLC.

miRNA	Hazard ratio	p value	Location	Gene references into functions
**miR-886-3p**	0.54	0.045	5q31.1	Predicting outcome in AML [43]; regulating cell proliferation and migration in familial non-medullary thyroid cancer [44].
**miR-150**	0.26	0.049	19q13.33	Down-regulated in leukemia (1), colorectal cancer [45] and malignant pancreatic tissues [35]; down-regulated associated with shorter survival and a worse response to adjuvant chemotherapy in colorectal cancer [37].

**Figure 2:**
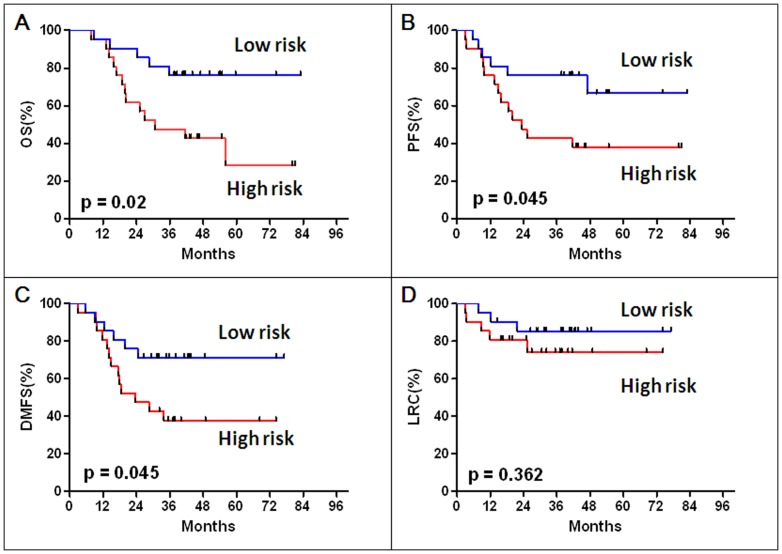
Kaplan-Meier analyses of overall survival (OS), progression-free survival (PFS), distant metastasis-free survival (DMFS) and local-regional control (LRC) according to the microRNA signature in the training data set (42 SCLC patients).

We then derived a formula to calculate the risk score for every patient from the expression level of the two miRNAs associated with OS, weighted by Cox regression coefficients: Risk Score  = (0.545 × expression level of miR-150) + (0.617 ×expression level of miR-886-3p). Patients in the training set were divided into high-risk or low-risk groups using the median miRNA signature risk score as the cutoff. Patients with higher risk scores are expected to have poor OS. Compared to patients with a low-risk miRNA signature, patients with a high-risk signature had significantly shorter median OS (12.6 months compared with not reached, p = 0.02) (Fig. 2A). Likewise, patients in the high-risk group had shorter PFS and DMFS times than those in the low-risk group (Both p-values were 0.045) (Fig. 2B-C). However, LRCs were similar between the two groups (p = 0.36) (Fig. 2D).

**Figure 3:**
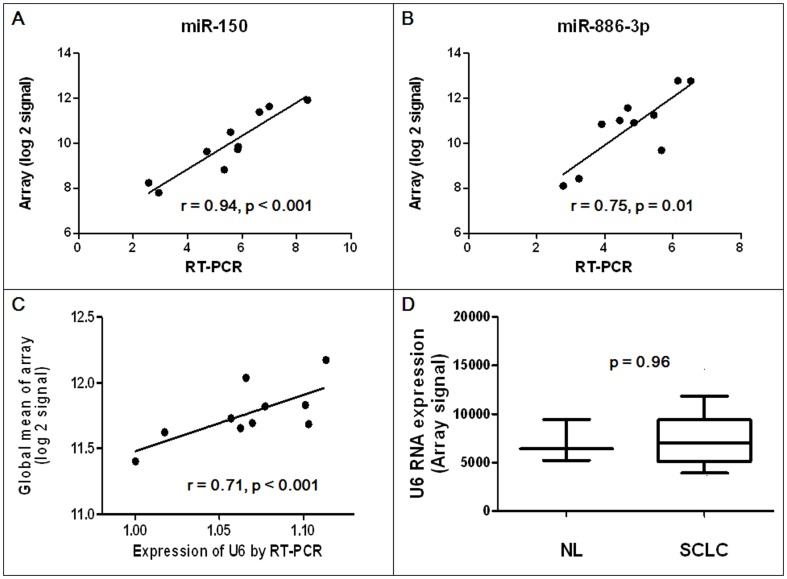
Correlation between RT-PCR Ct and log 2-transformed array signal intensity values for (A) miR-150 and (B) miR-886-3p. (C) Correlation between expression of U6 RNA by qRT-PCR and log 2-transformed global mean array expression. (D) Expression of U6 RNA in human normal lung (NL, n = 3) and small-cell lung cancer (SCLC, n = 42) samples by microarray method. Whiskers depict the 10 and 90 percentiles. p value is calculated by the Mann-Whitney U test.

To validate the microarray hybridization expression data, the expression levels of the two miRNAs associated with OS were quantified using qRT-PCR in 10 SCLC tissues randomly chosen from the training set. Significant correlation was observed between log 2-transformed array signal intensities and qRT-PCR Ct values for both miR-150 (r = 0.94, p<0.001) and miR-886-3p (r = 0.75, p = 0.01) (Fig. 3).

**Figure 4:**
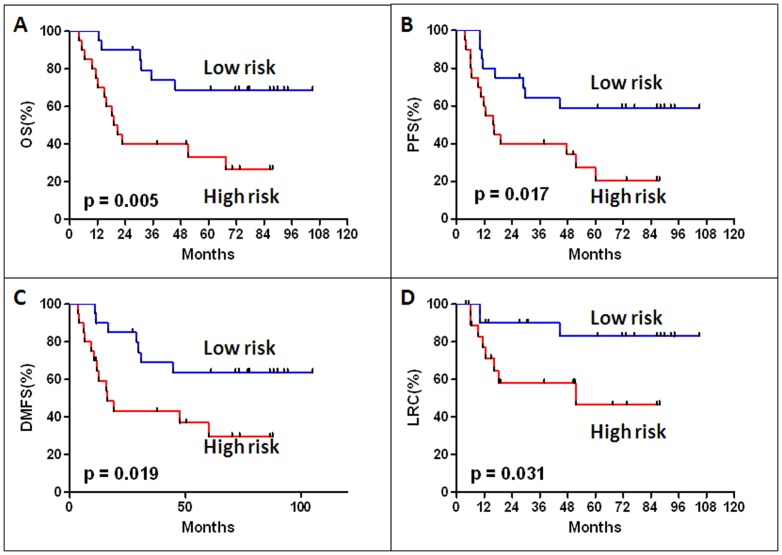
Kaplan-Meier analyses of overall survival (OS), progression-free survival (PFS), distant metastasisfree survival (DMFS) and local-regional control (LRC) according to the microRNA signature in the testing data set (40 SCLC patients).

Next, we used the same risk score formula obtained from the training set to calculate the miRNA signature risk score for 40 patients in the testing set, for which the miRNA levels in FFPE specimens were determined by qRT-PCR. Again, the high miRNA signature risk score patients exhibited shorter survival than in the low-risk group (median OS 18.9 months compared with not reached, p = 0.005) (Fig. 4A). PFS, DMFS, and LRC were also significantly shorter in the high than the in the low risk group (PFS p = 0.017, DMFS p = 0.019 and LRC p = 0.031, Fig. 4B, 4C, and 4D). The multivariate Cox-regression analysis was performed to further evaluate whether the dual-miRNA signature is an independent prognostic factor associated with survival in this testing set. Our results showed that the miRNA signature remained an independent prognostic factor for OS (HR: 0.26, 95%: CI 0.10–0.69, p = 0.007), PFS (HR: 0.36, 95%: CI 0.15–0.86, p = 0.02), DMFS (HR: 0.34, 95%: CI 0.13–0.86, p = 0.02), and LRC (HR: 0.26, 95%: CI 0.07–0.99, p = 0.05) after adjustment for age, gender and smoking status (Table 3).

**Table 3:** Multivariate Cox regression analysis of miRNA signature and survival in the testing set (N = 40).

Variable	Hazard Ratio	95%CI	p value
**Overall survival**			
miRNA signature	0.26	0.10–0.69	0.007
Age	1.96	0.77–5.02	0.16
Gender	1.12	0.11–11.71	0.92
Smoking status	0.57	0.07–4.49	0.60
**Progression-free survival**			
miRNA signature	0.36	0.15–0.86	**0.02**
Age	1.45	0.60–3.52	0.41
Gender	1.15	0.11–11.73	0.91
Smoking status	0.45	0.06–3.46	0.44
**Distant metastasis-free survival**			
miRNA signature	0.34	0.13–0.86	**0.02**
Age	1.48	0.57–3.84	0.42
Gender	0.84	0.07–9.83	0.89
Smoking status	0.50	0.07–3.89	0.51
**Local-regional control**			
miRNA signature	0.26	0.07–0.99	**0.05**
Age	2.38	0.66–8.54	0.19
Gender	0.52	0.04–6.90	0.62
Smoking status	1.34	0.15–11.67	0.79

## Discussion

In this study, we initiated a discovery phase exercise to identify key miRNAs by surveying the expression of 924 known miRNAs in FFPE specimens from 42 SCLC cases. We discovered that a dual-miRNA signature, miR-150 and miR-886-3p, was associated with OS and PFS. These findings were subsequently confirmed in an independent cohort of 40 SCLC patients using the qRT-PCR method, and the concordance between these two platforms was high.

Applying an optimal normalization method is particularly important for miRNA profiling to minimize technical variations and to get meaningful biological changes. However there is no consensus normalization method and recommendations at present for either microarray or qRT-PCR methods in terms of miRNA profiling. In this study, we used a global normalization method in the training set, which is considered more suitable for large-scale miRNA-profiling data sets [27]. In the testing set, we used U6 RNA for normalizing data because it is the most frequently reported reference gene for miRNA expression studies in lung cancer in the literature [21], [28]–[30]. We also correlated the expressions of the two miRNAs on qRT-PCR with array expressions, and confirmed that different miRNA-profiling methods did not influence the results of miR-150 and miR-886-3p expressions (Fig.3A-B). In addition, the correlation of U6 expression on qRT-PCR with global mean array expression was observed (r = 0.71, p<0.001; Fig. 3C). U6 RNA showed absolute fold changes lower than 1.2-times and had no significant differences between the NL and SCLC samples (p = 0.96, Fig. 3D).

Currently, there are only two published studies examining miRNA expression in human SCLC samples [31], [32]. Lee et al. investigated the expression of a panel of seven miRNAs (miR-21, miR-29b, miR-34a/b/c, miR-155, and let-7a) in 31 SCLC tumors [31]. They reported that the expression of these seven miRNAs was unrelated to the clinical characteristics of SCLC patients and was not prognostic in terms of OS or PFS, nor was the expression level predictive of treatment response. These data were consistent with our findings. Ranade and colleagues evaluated the expression of 880 validated human mature miRNAs with an additional 473 validated human pre-miRNAs in 34 formalin-fixed, paraffin-embedded SCLC tumor specimens and found that miR-92a-2* was independently associated with survival. Although this study showed for the first time that miRNAs can be prognostic for SCLC, it included cases with both limited stage (12.1%) and extensive stage (87.9%) cancer at diagnosis, and patients with extensive-stage disease have cancers that are considered more heterogeneous. Furthermore, in this study, the tumor tissues were collected not only from primary tumors but also from lymph nodes and distant metastases, which would confound the evaluation results because miRNA expression is considered to be tissue specific. In addition, only half of the patients in this study received radiation during first-line therapy. This is important because radiation might significantly affect survival in both limited-stage [33] and extensive-stage SCLC [34]. To date, our study is the first showing that in early stage SCLC patients treated with surgery and adjuvant chemotherapy, a miR-150/miR-886-3p signature predicts OS and PFS in both training and testing sets. This association was independent of age, gender and smoking status. These findings indicate that the miR-150/miR-886-3p signature might be a good candidate as a molecular prognostic marker and would potentially help doctors to identify patients at high-risk who may benefit from more extensive adjuvant therapy.

We also found that both miR-150 and miR-886-3p were negatively associated with OS. Comparing the miRNA levels between NL and SCLC tissues also verified that miR-150 and miR-886-3p expression levels in SCLC were much lower than in NL samples. Because of the small NL sample size, we were unable to identify a pattern of persistently small p values. However, these data consistently suggested that miR-150 and miR-886-3p might serve as important tumor suppressors in the development and progression of SCLC and might function with potential anticancer targets. Our previous experimental study has demonstrated a novel miR-886-3p-mediated mechanism underlying the aberrant expression of in polo-like kinase 1 (PLK1) and transforming growth factor beta 1 (TGF-β1) in SCLC [23]. The 3′ untranslated regions of PLK1 and TGF-β1 contain highly conserved miR-886-3p binding motifs, and direct interactions with miR-886 down-regulates endogenous PLK1 and TGF-β1 protein levels. We also showed that miR-886-3p over-expression inhibited proliferation, migration and invasion in SCLC cells, as well as suppressing the growth and metastasis of SCLC xenografts in nude mice. Finally, we revealed that the mechanism behind the down-regulated expression of miR-886-3p in SCLC was mediated by DNA hypermethylation of its promoter. Altogether, these findings establish a miR-886-3p-PLK1/TGF-b1 nexus as a novel regulator and promising therapeutic target for SCLC.

The observation of miR-150 as a tumor suppressor in SCLC is consistent with similar observations in pancreatic cancer [35], liver cancer [36], colorectal cancer [37], NSCLC [38], malignant lymphoma [39] and acute leukemia [40]. However, its involvement in SCLC has not been reported, and the precise molecular mechanisms behind its altered expression are unclear. Jiang et al. recently found that miR-150 maturation is repressed by MYC in MLL-associated leukemia. In addition, miR-150 is reported to be a protective miRNA that directly targets c-Myb and other factors [36], [40]. miR-150 interacts with the 3′UTR of c-Myb mRNA, and over-expression of miR-150 down-regulates c-Myb protein levels [36]. Interestingly, both MYC family members and the c-Myb gene are two well-known and dominant proto-oncogenes that are frequently found to be amplified or over-expressed in SCLC [41]. Taken together, these recently published data together with ours, suggests that miR-150 has a tumor suppressor effect in SCLC because low levels of miR-150 expression was correlated with poor survival. However, additional studies are needed to elucidate the molecular mechanisms of both the cause and effect of altered miR-150 expression in the development and progression of SCLC.

Limitations of this study include its relatively small sample size, a necessity because only a small number of patients (less than 5%) with early peripheral disease [42] are surgical candidates. More than eight hundred SCLC patients were treated with chemotherapy at our hospital during the same time period. As a result, biomarker analyses from SCLC patients are reported much less frequently compared with NSCLC patients. Additionally, because of this fact, multi-test corrections were not applied in this study because these corrections prevent type I errors at the cost of a rise in type II errors. Nevertheless, the correlation between the miRNA signature and the clinical outcomes were independently confirmed in a second patient set. Larger-scale studies are needed to confirm our findings. Another limitation is the retrospective design. However, all measurements were performed in a blind manner. Finally, the potential interaction between miR-150 and miR-886-3p should be further investigated to elucidate the mechanism behind the prognostic role of this dual-miRNA signature.

In conclusion, our preliminary results suggested that the miR-150/miR-886-3p signature might predict cancer progression and survival in early stage SCLC patients and might be a promising prognostic biomarker and potential therapeutic targets for SCLC patients.
